# RNAseq of INOCA patients identifies innate, invariant, and acquired immune changes: potential autoimmune microvascular dysfunction

**DOI:** 10.3389/fcvm.2024.1385457

**Published:** 2024-06-24

**Authors:** Kevin Jaatinen, Palak Shah, Ramesh Mazhari, Zane Hayden, Richard Wargowsky, Tisha Jepson, Ian Toma, John Perkins, Timothy A. McCaffrey

**Affiliations:** ^1^ Department of Medicine, Division of Genomic Medicine, The George Washington University, Washington, DC, United States; ^2^ INOVA Heart and Vascular Institute, Fairfax, VA, United States; ^3^ Department of Medicine, Division of Cardiology, The George Washington University, Washington, DC, United States; ^4^ The St. Laurent Institute, Woburn, MA, United States; ^5^ True Bearing Diagnostics, Washington, DC, United States; ^6^ Department of Clinical Research and Leadership, The George Washington University, Washington, DC, United States; ^7^ Department of Microbiology, Immunology, and Tropical Medicine, The George Washington University, Washington, DC, United States

**Keywords:** INOCA, RNAseq, MAIT (mucosal-associated invariant T) cell, coronary artery disease, T cell, neutrophil, plasmacytoid dendritic cell, invasive coronary angiography (ICA)

## Abstract

**Background:**

Ischemia with non-obstructive coronary arteries (INOCA) is a major clinical entity that involves potentially 20%–30% of patients with chest pain. INOCA is typically attributed either to coronary microvascular disease and/or vasospasm, but is likely distinct from classical coronary artery disease (CAD).

**Objectives:**

To gain insights into the etiology of INOCA and CAD, RNA sequencing of whole blood from patients undergoing both stress testing and elective invasive coronary angiography (ICA) was conducted.

**Methods:**

Stress testing and ICA of 177 patients identified 40 patients (23%) with INOCA compared to 39 controls (stress-, ICA-). ICA+ patients divided into 38 stress- and 60 stress+. RNAseq was performed by Illumina with ribosomal RNA depletion. Transcriptome changes were analyzed by DeSeq2 and curated by manual and automated methods.

**Results:**

Differentially expressed genes for INOCA were associated with elevated levels of transcripts related to mucosal-associated invariant T (MAIT) cells, plasmacytoid dendritic cells (pcDC), and memory B cells, and were associated with autoimmune diseases such as rheumatoid arthritis. Decreased transcripts were associated with neutrophils, but neutrophil transcripts, *per se*, were not less abundant in INOCA. CAD transcripts were more related to T cell functions.

**Conclusions:**

Elevated transcripts related to pcDC, MAIT, and memory B cells suggest an autoimmune component to INOCA. Reduced neutrophil transcripts are likely attributed to chronic activation leading to increased translation and degradation. Thus, INOCA could result from stimulation of B cell, pcDC, invariant T cell, and neutrophil activation that compromises cardiac microvascular function.

## Graphical Abstract

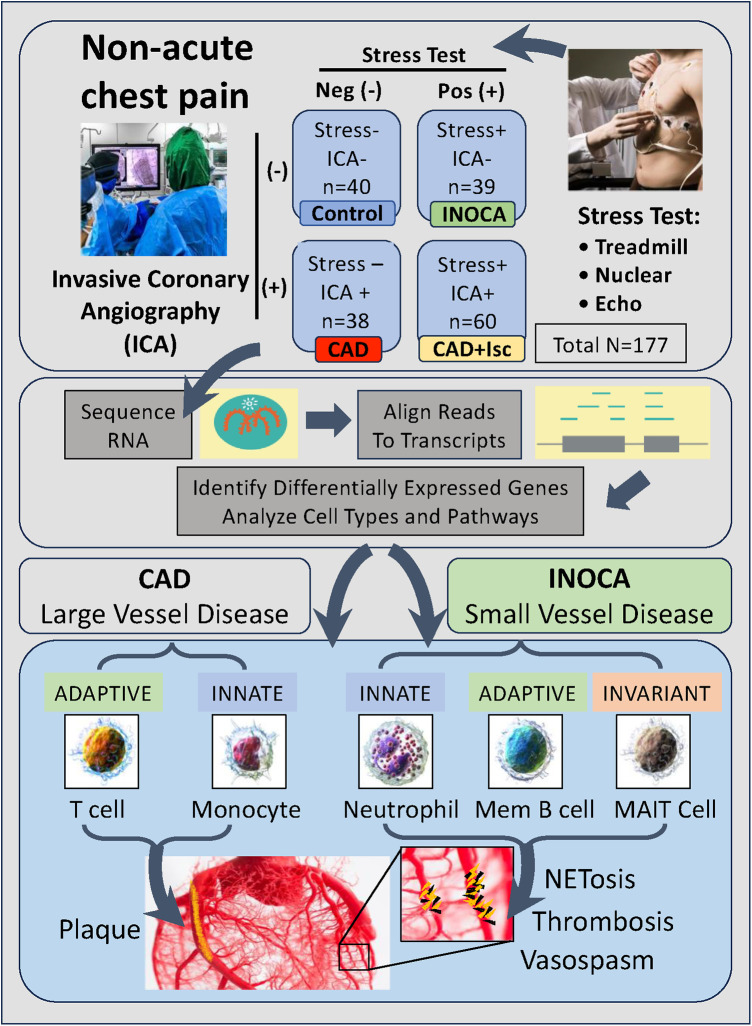

## Highlights

• INOCA is associated with elevated levels of transcripts relevant to the acquired immune system, especially memory B, plasmacytoid dendritic cells, and MAIT cell lineages, and a reciprocal decrease in transcripts associated with the innate immune system, particularly neutrophils.
• The INOCA profile has significant differences with the CAD profile. The INOCA transcripts affected are of relatively high absolute abundance and some have high fold-changes in the acquired immune lineages, compared to the relatively low level and low fold changes observed for CAD in the T regulatory lineage.
• The inclusion of INOCA patients in prior “control” groups for CAD likely masked some significant transcripts that are shared by both INOCA and CAD.
• Despite the innate immune system involvement, INOCA likely does not involve an active bacterial, biofilm, or viral infection.
• The RNA profile is consistent with an indolent autoimmune syndrome, leading to chronic, low-grade activation of NETosis, with one of the targets being the coronary microvasculature.

## Clinical perspectives

• Patients presenting with non-acute, stable chest pain, and demonstrating ischemia upon stress testing, are often found not to have obstructive coronary arteries, termed Ischemia with Non-Obstructive Coronary Arteries (INOCA).
• One explanation for INOCA is that these patients have coronary microvascular disease (CMD), or vasospasms, which compromises perfusion, but is not readily detectable by angiography.
• The present studies employed state-of-the-art RNA sequencing of whole blood RNA to identify similarities and differences in the circulating transcriptome between CAD and INOCA.
• The INOCA patients demonstrated a substantially different profile that was dominated by changes in transcripts related to neutrophils, plasmacytoid dendritic cells, mucosal-associated invariant T cells, and memory B cells.
• By comparison, CAD patients, in this, and other studies, consistently show transcriptome changes related principally to T cells, especially T regulatory cells, that are decreased in numbers.
• This suggests that while both CAD and INOCA have immune components, they are likely mediated by different types of cells, and thus may have different initiating events, and require different therapeutic approaches.

## Introduction

Ischemia with non-obstructive coronary arteries (INOCA) is an important clinical entity involving coronary microvasculature dysfunction (CMD) and/or vasospasm of the coronary arteries or arterioles. INOCA is estimated to affect 3–4 million in the U.S. with more women than men affected (1). INOCA likely affects 20%–30% of patients presenting with non-acute chest pain. These patients typically undergo a series of tests that include an ECG stress test on a treadmill, with or without nuclear imaging, possibly ultrasound imaging, and potentially invasive coronary angiography (ICA) or CT angiography (CTA). INOCA is related to and could progress to angina with no obstructive coronary arteries (ANOCA, aka cardiac syndrome X, CSX) and/or myocardial infarction NOCA (MINOCA). Women with INOCA demonstrate elevated ultra-high sensitivity troponin I levels, suggesting chronic, low grade cardiomyocyte damage (2).

The absence of angiographic stenosis in these ischemic patients is consistent with poor microvascular perfusion, such as CMD, and/or vasospasm of larger arteries and arterioles. About half of systemic lupus erythematosus (SLE) patients with suspected INOCA demonstrate CMD (3). In patients with ANOCA, acetylcholine provocation indicated that ANOCA was caused by CMD (33%) or epicardial artery vasospasm (26%), and was more prevalent in women than men (4). Upon invasive angiography for stable chest pain, women are much more likely to demonstrate normal coronary arteries (40.2%) than men (16.1%) (5).

Both MINOCA and INOCA are related to poor long-term outcomes, including heart failure (6). Major trials are underway, such as the WARRIOR trial, to compare aggressive interventions to slow or reverse the progression of INOCA in women (7). Despite the substantial clinical impact, there is only a rudimentary understanding of the mechanism of INOCA, and thus, the present studies were undertaken to generate new hypotheses about the pathways involved.

## Methods

### Experimental design

**Figure 1:**
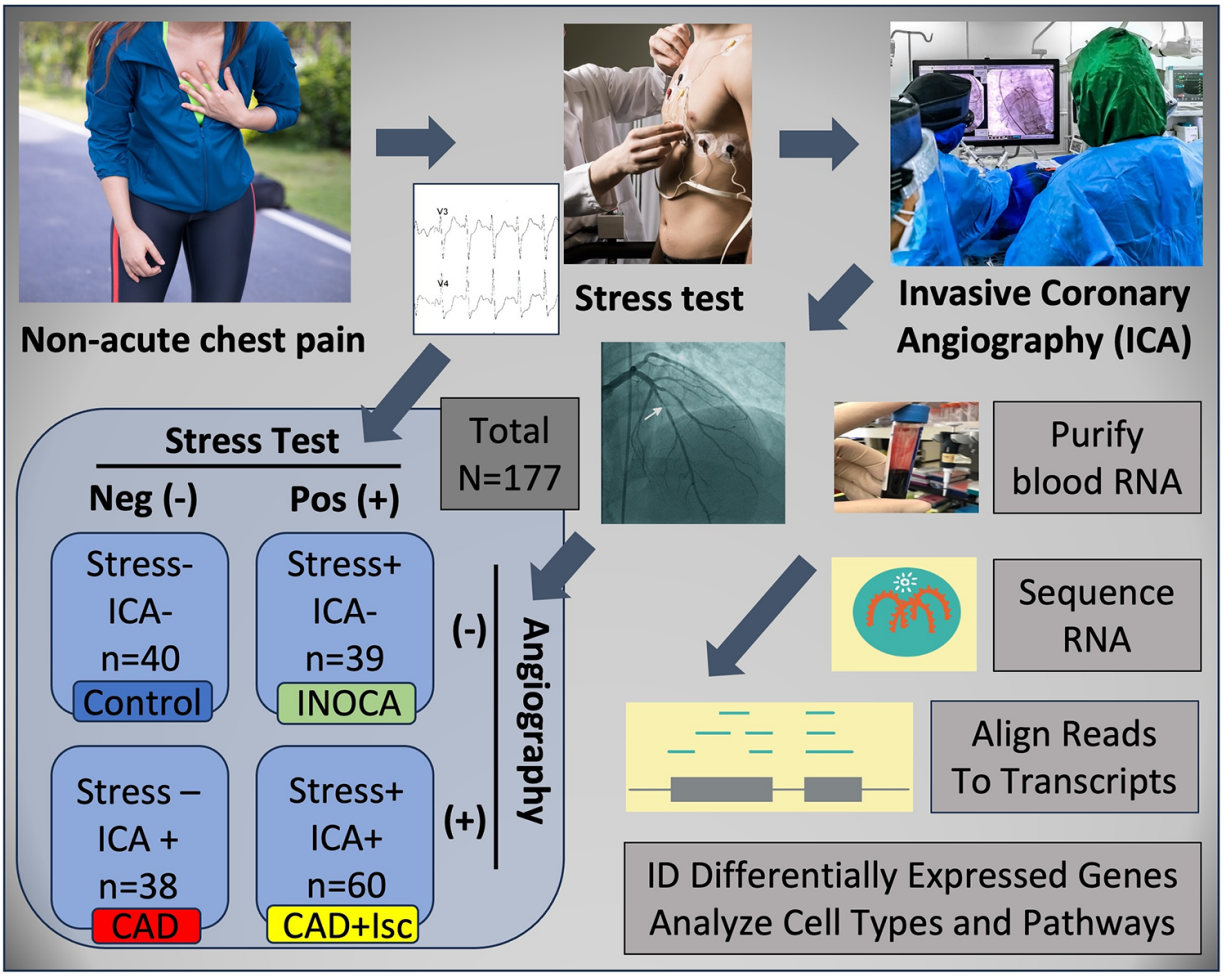
Schematic of study design. Patients presenting with non-acute chest pain can have several underlying disorders, including CAD. Patients referred for elective invasive coronary angiography (ICA) that had prior stress tests were candidates for the current study. On the basis of the stress test and ICA results, patients were categorized into Control (normal stress test and no coronary stenosis ≥20%) or INOCA (abnormal stress test and no coronary stenosis ≥20%). Prior to ICA, a blood sample was drawn into Tempus blood RNA stabilizer, and frozen at −80°C until it was analyzed by Illumina Total RNAseq. The total reads were filtered, aligned to the human genome, and then counted into all known transcripts, and compared between groups to identify differentially expressed genes (DEGs), and the cell types and pathways involved.

INOCA was defined as a positive (+) stress test (= ischemia), with no CAD at ICA (<20% stenosis). A positive stress test included abnormal functional studies including nuclear stress test, or stress echocardiography, with evidence of reversible ischemia on a nuclear study or reversible stress-induced wall motion abnormality on stress echocardiography, as determined by the attending cardiologist according to current ACC/AHA guidelines (8). The primary analysis compared the stress+ CAD- INOCA groups to control patients that were defined as stress- and CAD-. Invasive coronary angiography (ICA) was used to identify patients with obstructive CAD vs. those without CAD (Figure 1). Blood was collected in Tempus RNA stabilizing tubes prior to ICA, stored at −80°C and then profiled by RNAseq as described below. Coronary angiograms were digitally interpreted by the attending cardiologist. For statistical power, and a conservative definition of “no CAD”, initial analyses compared LOW CAD (<20% stenosis) to CAD combined (>20% stenosis) yielding groups of similar size.

### Patients

Patients presenting for ICA related to non-emergent complaints of typical or atypical chest pain, exertional dyspnea, or other symptoms suggestive of CAD, provided written, informed consent for participation in this study under a protocol approved by The George Washington University IRB and the INOVA Fairfax IRB. Patients with heart failure, non-ST segment elevation myocardial infarction (MI) and ST elevation MI (STEMI) were excluded from the study. All patients presented in the period of October 2017 through September 2019, prior to the COVID19 pandemic. The design of the study is shown schematically in Figure 1. Patients admitted for ICA had three Tempus blood RNA tubes collected by peripheral venipuncture or an indwelling catheter just prior to ICA, which was typically up to 2 weeks after stress testing. After blood sampling, these studies were purely observational and did not alter the patient's clinical course. All relevant clinical data was captured for comparison to the transcriptome profiling.

### Clinical risk factor assessment

Cardiac medical histories were evaluated by their attending cardiologists to determine CAD risk factors, according to the 2013 ACC/AHA Guidelines on the Assessment of Cardiovascular Risk (9). Hypertension was defined as a history of blood pressure ≥140/90 mmHg and/or treatment with anti-hypertensive medications. A family history of CAD was determined by MI or cardiac death in a first-degree relative. Diabetes mellitus was indicated by fasting glucose of ≥126 mg/dl and/or use of insulin or oral hypoglycemic agents. Current smoking was defined by active smoking within the most recent 3 months. Dyslipidemia was judged by the guidelines of National Cholesterol Education Program Adult Treatment Panel III or by treatment with lipid lowering medication.

### Transcriptome profiling

#### RNA processing

Tempus stabilized frozen (−80°C) peripheral blood samples were thawed and RNA was isolated using Tempus Spin RNA Isolation Kits (ThermoFisher Scientific) according to the manufacturer's protocol. The total nucleic acid isolate was treated with 4 Units of DNAse (Turbo DNA-free Kit, Ambion). The typical nucleic acid yield from 3 ml Tempus blood tubes averaged ∼3 µg, with an RNA integrity (RIN) score >7 (10 is maximal) on Agilent 2100 Bioanalyzer (Table 1).

**Table 1:** Demographic, angiographic, and analytical parameters of the patients.

Angiographic stenosis >20% →	CAD negative		CAD positive		Overall
Stress test →	Negative (-)	Positive (+)	Negative (-)	Positive (+)	
Sample size	40	39	38	60	177
Sex (male)	26 (65.0%)	21 (53.8%)	22 (57.9%)	42 (70.0%)	111 (62.7%)
Age (years)	64.0 (8.61)	63.2 (12.2)	74.1 (11.4)*	66.1 (10.5)	66.7 (11.4)
Race (White/Caucasian)	33 (82.5%)	30 (76.9%)	34 (89.5%)	50 (83.3%)	147 (83.1%)
BMI	32.2 (10.6)	30.8 (8.14)	28.5 (5.95)	31.0 (8.27)	30.7 (8.43)
% Stenosis	2.43 (4.68)*	3.49 (5.39)*	58.4 (25.6)*	73.3 (25.2)*	38.7 (37.8)
Systolic blood pressure (mmHg)	128 (18.7)	131 (17.9)	136 (26.4)	138 (25.3)	134 (23.0)
Diastolic blood pressure (mmHg)	72.2 (12.6)	71.3 (9.42)	70.2 (11.6)	75.9 (13.3)*	72.8 (12.1)
Ejection fraction (%)	55.9 (10.5)	55.5 (10.7)	60.2 (8.52)*	55.7 (11.5)	56.7 (10.6)
Creatinine (mg/dl)	0.965 (0.250)	0.950 (0.244)	1.07 (0.420)	1.04 (0.342)	1.01 (0.325)
Dyslipidemia	23 (57.5%)	20 (51.3%)	14 (36.8%)	25 (41.7%)	82 (46.3%)
Statin use	21 (52.5%)	15 (38.5%)	24 (63.2%)	45 (75.0%)	105 (59.3%)
Diabetes mellitus	30 (75.0%)	32 (82.1%)	26 (68.4%)	41 (68.3%)	129 (72.9%)
Smoking (never)	27 (67.5%)	24 (61.5%)	22 (57.9%)	29 (48.3%)	102 (57.6%)
RNA yield (ug/3 ml blood)	3.19 (0.89)	3.13 (0.75)	3.29 (0.93)	3.21 (0.94)	3.20 (0.88)
Unique mapped reads (million)	14.9 (3.9)	14.6 (4.1)	14.1 (5.2)	14.6 (4.4)	14.6 (4.4)

* Indicates *p* < 0.05 by *t* test, uncorrected for multiple testing.

#### RNA sequencing

Total RNA, post-DNAse, was sequenced using the Illumina TruSeq Stranded Total RNA sequencing kit, which includes depletion of ribosomal RNA (rRNA) by Ribo-Zero rRNA Removal Kit (Illumina). Each RNAseq run was composed of 24 patient blood RNAs barcoded for multiplexing onto the NextSeq 500 using the High-Output 2 × 75 bp kit. The resulting 150 bp paired end reads were parsed to each barcode/patient, concatenated across the 4 read chambers, trimmed, and then aligned to the HG38 genome using STAR aligner (version 2.5.2b) (10). Using quantMode Gene Counts option, STAR counted the number of reads per gene while mapping. Differentially expressed genes (DEGs) were identified by using raw read counts compared between groups with DESeq2 (11). Absolute expression levels are reported as DeSeq2 normalized read counts (nRC).

#### Cell type analysis

The blood-borne cell types affected by INOCA were examined by two different approaches using precurated lists of transcripts with preferential expression in specific cell types, as determined by RNAseq of sorted cell populations, and single cell RNAseq, as a part of the Human Protein Atlas Project (12). First, the transcripts from the Control vs. INOCA DEG list were cross-referenced to the expression of those transcripts in the 19 immune cell types quantified in the Blood Cell portion of the Human Protein Atlas. The transcripts upregulated and downregulated in INOCA were considered separately in case they represented different cell types. A mean normalized expression of all up- or down-regulated was calculated for each cell type. A second approach was to use pre-defined cell-specific transcripts unrelated to the DEGs, typically the top 10 per cell type, identified by the Blood Atlas for each immune cell type, and then compute the normalized mean expression in the Control vs. INOCA patients.

#### Statistical methods

Continuous data, such age, blood pressure or ejection fraction was compared by Student's *t* test. Categorical data, such as sex, were compared with the chi-square test. Gene expression levels were treated as a continuous variable that is not normally distributed, and thus were compared by Mann-Whitney *U*. Intercorrelations of gene expression levels were calculated with Spearman's *r*.

## Results

### Patient characteristics

As expected from patients undergoing elective ICA (*n* = 177), there were a significant percentage that did not have CAD > 20% (*n* = 79, 44.6%), which is consistent with nationwide rates for “no CAD” elective catheterizations (13). Of these 79 “no CADs”, there were more males overall (59.5%) than females, but were represented about equally in the stress+ and stress- groups (Table 1). The other demographic and lab values, such as age, race, BMI, blood pressure, diabetes, ejection fraction, and creatinine were not different between groups, except that stress-/CAD+ patients were significantly older and had higher ejection fractions. Likewise, the RNA yield (∼3.1 µg/3 ml blood) and the number of unique mapped reads (∼15 million reads per patient), were not different between groups.

### Differentially expressed genes in INOCA patients

**Figure 2:**
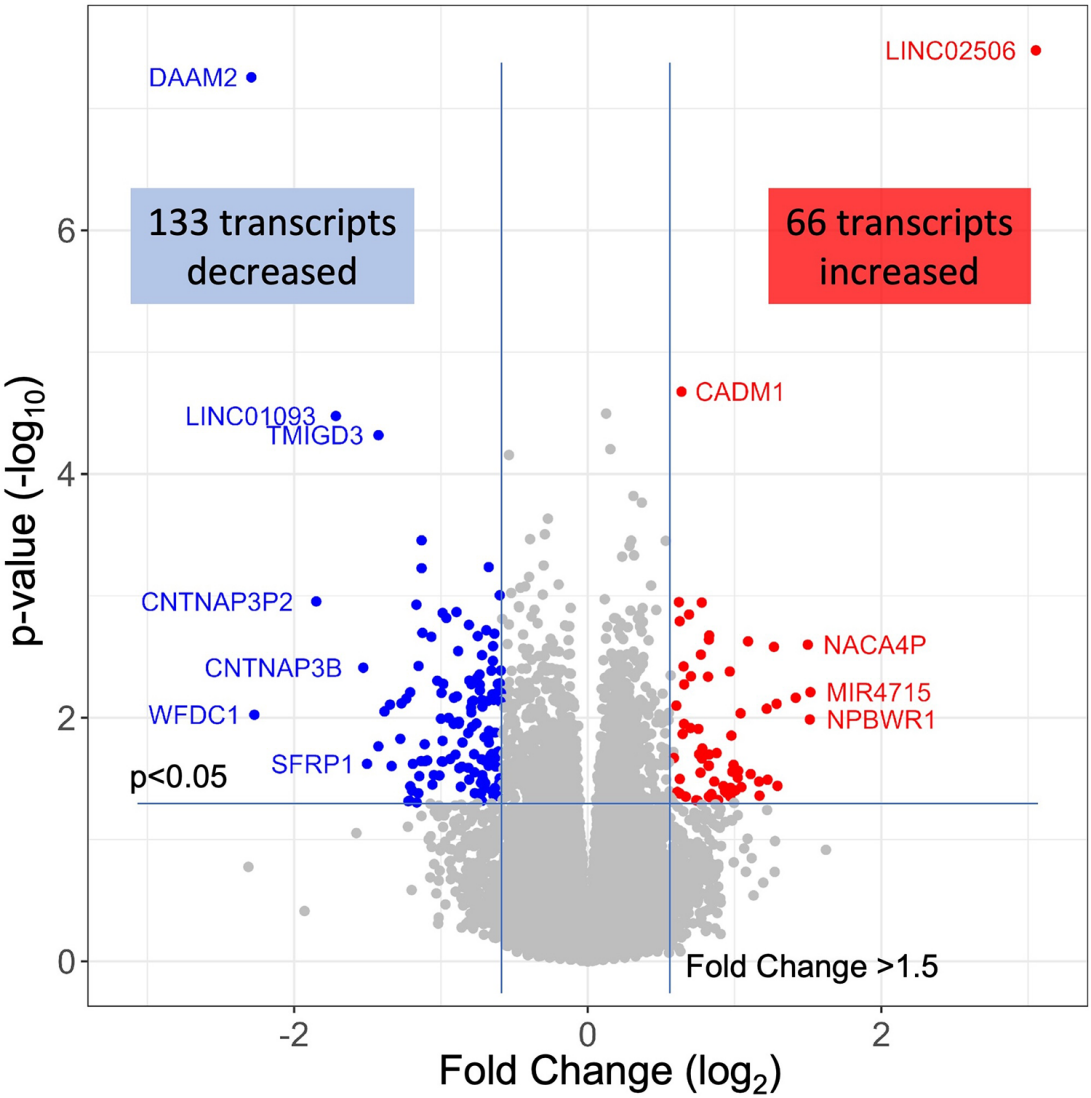
RNAseq analysis of INOCA patients. A total of ∼28 K transcripts were quantified and averaged within groups of control or INOCA patients. Each transcript is plotted as a function of the fold difference between groups (*X*-axis, log_2_ scale) vs. the *p*-value of the difference computed by DeSeq2 (*Y* = axis, log_10_ scale). Transcripts below a 0.05 uncorrected *p*-value and also >1.5-fold change are indicated as increased (Red) or decreased (Blue), with selected transcripts named by gene symbol.

The 79 patients without coronary stenosis >20% divided about equally into 40 patients that had a normal stress test, and 39 patients with an abnormal stress test indicative of ischemia, and thus INOCA. The RNAseq expression values were compared between groups using DeSeq2 to identify transcripts of potential interest. DeSeq2 identified 1,581 transcripts that were differentially expressed at an uncorrected *p*-value of 0.05 (Supplementary Data S1). This list was further filtered to include only transcripts that showed a greater than 1.5-fold increase or decrease in INOCA, yielding 66 transcripts increased, and 133 decreased (199 total, Supplementary Data S1). Only 2 transcripts, DAAM2 (down 4.9-fold) and LINC02056 (up 8.3-fold) were significant using the Bonferroni corrected *p*-value of 0.05, irrespective of fold-change (Figure 2). These 199 transcripts were used to understand the nature of the transcriptome changes in INOCA.

### Annotation of differential expressed transcripts in INOCA patients

The primary list of 199 INOCA transcripts was analyzed by both manual and automated methods to understand the types of transcripts that were modulated. The transcripts were extensively analyzed by both automated (i.e., GeneCards, NIH DAVID, Ingenuity Pathways) and manual methods (i.e., PubMed) that considered: the direction of the change, the type of RNA involved (coding RNA/mRNA, microRNA, non-coding RNA, etc.), the cell types, functional pathways, annotations, and disease associations. Selected transcripts are shown in Tables 2, 3. Many of the miRNA and ncRNAs are poorly annotated and thus will require further study to understand their involvement. Focusing on the mRNAs, a clear pattern emerged: the upregulated transcripts fell into 2 groups, one associated with mucosal-associated invariant T (MAIT) cells and the other with B cells, especially memory B cells (Table 2). In many cases, the memory B cell transcripts could also be expressed by plasmacytoid dendritic cells (pcDC).

**Table 2:** Selected transcripts elevated in INOCA patient whole blood RNA.

Upregulated transcripts				Cell type	Pathway	Disease association	Annotation
Gene	Level	INOCA	CAD				
Symbol	(nRC)	Change	Change				
AGER	2.33	1.71	1.97	Low all	Inflammation, glucose	Diabetes, regulates T cell activation	Advanced glycosylation end-product specific receptor
ARSJ	1.03	1.82	1.61	MAIT T	arsenic efflux, hypoxia	Body height, ceramide	Arylsulfatase family member J
CADM1	11.18	1.56	1.03	gdT, MAIT, Treg, myDC	Adhesion IgG family	Distinguishes antitumor from autoimmune T cell	Cell adhesion molecule 1
CFH	38.53	1.54	1.23	MAIT T	Complement	Modulates atherosclerositic process in mouse model	Complement factor H
DKK2	0.84	1.96	1.36	MAIT T	Wnt and Notch	Aortic dissection	dickkopf WNT signaling pathway inhibitor 2
DMKN	1.67	1.96	1.73	MAIT T, NK, memB	Erk, STAT3	T2DM	dermokine (DMKN), transcript variant 5
ERFE	2.15	2.13	3.48	Low all	C1qTNF pathway	Erythropoesis and iron metabolism, CAD	Erythroferrone
ESM1	3.01	1.76	1.24	MAIT T, etc	Akt, VEGF	Immune signature in cancers	Endothelial cell specific molecule 1
HID1	6.25	1.54	1.02	pcDC, T, MAIT, gdT	Vesicles	Syndromic encephalopathy	HID1 domain containing
NME2	0.39	2.16	1.70	MAIT, others, low PMN	Stemness, apoptosis	Regulates class switch recombination in B cells	NME/NM23 nucleoside diphosphate kinase 2
NTN4	4.19	1.57	−1.05	MAIT T	Wnt B-Catenin	Immune signature in cancers	Netrin 4
CCL8	0.43	2.25	1.46	Monocyte, memB	Chemoattractant	IBS	C-C motif chemokine ligand 8
CPXM1	0.83	2.06	2.05	memB, DC, NK	Glucose metab	Crohns inflamm	Carboxypeptidase X, M14 family member 1
FA2H	0.57	2.44	2.23	Only memB	Sphingolipids	Psoriasis, atopic derm, leukodystrophy, dystonia	Fatty acid 2-hydroxylase
GLDC	4.24	1.97	−1.32	Mainly memB, PMN	Mitochondrial	Immune infiltration in diabetic ulcers	Glycine decarboxylase, nuclear gene for mitochondrial product
IGLL5	128.22	1.58	−1.08	Only memB, B	Immunoflobin	Marker in Crohns with MZB1 JCHAIN, RA hub gene	Immunoglobulin lambda like polypeptide 5
JCHAIN	293.81	1.78	−1.27	B cell	IgA and IgM	Marker of autoimmune, marker Crohns w MZB1, IGLL5	Joining chain of multimeric IgA and IgM
MZB1	25.52	1.71	−1.12	Only memB, pcDC	Promotes JCHAIN	Marker in Crohns w JCHAIN IGLL5	Marginal zone B and B1 cell specific protein
TNFRSF17	8.99	1.77	−1.19	memB, B, pcDC	B-Cell Maturation	Marker of PC in autoimmune w JCHAIN	TNF receptor superfamily member 17, B-Cell Maturation Factor

Cell type abbreviations: MAIT T, mucosal-associated invariant T cell; NK, natural killer cell; memB, memory B cell; pcDC, plasmacytoid dendritic cells; gdT, gamma-delta T cell; DC, dendritic cell; PMN, polymorphonuclear leucocyte/neutrophil.

**Table 3:** Transcripts decreased in INOCA patient whole blood RNA.

Down regulated transcripts				Cell type	Pathway	Disease association	Annotation
Gene	Level	INOCA	CAD				
Symbol	(nRC)	Change	Change				
LCN2	81.85	−1.53	1.05	Neutrophil	Transport, iron	Stroke, many others	Lipocalin 2
CRISP3	36.34	−1.54	1.09	Neutrophil	Transport	Immune thrombocytopenia, eosinophilic esophagitis	Cysteine rich secretory protein 3
ABCA13	119.65	−1.57	1.11	Basophil, neutrophil	Transport, cholesterol	Alcohol & diabetes Framingham	ATP binding cassette subfamily A member 13
MCEMP1	61.56	−1.68	1.00	Mono, neutrophil	Activation, adhesion	Septic immune cardiomyopathy, AAA with KCNE1 CLEC4D, stroke	Mast cell expressed membrane protein 1
CEACAM8	60.14	−1.62	1.10	Neutrophil	Activation, adhesion	Septic immune cardiomyopathy	Carcinoembryonic antigen cell adhesion molecule 8, CD66b
INHBA	4.02	−1.99	−1.35	Neutrophil, mono	TGF-ß, differentiation	T cell suppressor	Inhibin subunit beta A, activin beta A-chain
MAK	162.97	−1.56	1.03	Neutrophil	Interferon response	Response to IFN, retin pigmentosa	Male germ cell associated kinase
ZBTB16	264.33	−1.57	−1.16	MAIT GDT	Antiinflammatory, reg by lncANRIL	Con Ht Dis allele sp expr; PCOS; MS; AAA; metabolic syndrome;	Zinc finger and BTB domain containing 16
CLEC4E	760.50	−1.60	−1.03	Neutrophil, mono	Adhesion	AAA w KCNE1 MCEMP1, STEMI biomarker	C-type lectin domain family 4 member E
KCNE1	50.16	−1.64	−1.01	eos, baso, neut mono	Transport	AAA w CLEC4E, MCEMP1	Potassium voltage-gated channel E subunit 1
MCEMP1	61.56	−1.68	1.00	Mono, neutrophil	Activation, adhesion	AAA w KCNE1 CLEC4D, stroke, sepsis, septic cardiomyopathy	Mast cell expressed membrane protein 1
DAAM2	100.53	−4.90	1.14	Neutrophil only	Wnt, enable actin binding, GTPase	Nephrotic syndome, body height, osteoporosis, schizophrenia	Dishevelled associated activator of morphogenesis 2
PFKFB3	732.93	−1.55	−1.01	Neutrophil mono	HIF, fructose, glycolytic	Hypoxia, sepsis,	6-phosphofructo-2-kinase/fructose-2,6-biphosphatase 3
IL17RB	1.70	-1.88	-1.17	baso, Treg, Tcell	Interleukin 25 receptor	Inflammation	Interleukin 17 receptor B
TLR2	2,923.94	−1.51	−1.01	Neutrophil, mono	Toll like receptor	LPS receptor triggering activation	Toll like receptor 2
IL18RAP	886.41	−1.51	−1.13	Neut, NK, MAIT, T cell	IL18 receptor	Atopic dermatitis, Crohn's, Lupus, MI Risk, Celiac	Interleukin 18 receptor accessory protein
SFRP1	1.11	−2.84	1.86	Low all	Wnt	Modulates inflammation	Secreted frizzled related protein 1
NPC1L1	0.52	−2.61	−2.62	Low all	Transport, cholesterol		NPC1 like intracellular cholesterol transporter 1
NPM2	1.31	−1.84	−2.19	Eosinophil	Histone,		Nucleophosmin/nucleoplasmin 2
PDZD7	2.15	−1.63	−1.88	Weak neutrophil	Cilia, binds CADM1	Autosomal recessive deafness	PDZ domain containing 7

Cell type abbreviations: MAIT T, mucosal-associated invariant T cell; Mono, monocyte; eos, eosinophil; baso, basophil; Treg, T regulatory cell; NK, natural killer cell; memB, memory B cell; pcDC, plasmacytoid dendritic cells; gdT, gamma-delta T cell; DC, dendritic cell; PMN, polymorphonuclear leucocyte/neutrophil.

Conversely, the down-regulated transcripts were often strongly indicative of the granulocyte class of innate immune cells: basophils, eosinophils, and neutrophils (Table 3). Some of these mRNAs are considered consensus markers for neutrophils: CEACAM8 (aka CD66b) and CEACAM6 (CD66c), bacterial permeability increasing protein (BPI), CLEC4E, and TLR2. CEACAM8/CD66b, for instance, is a surface marker for neutrophils, similar to CD15, that is commonly used by our group, and many others, to identify and isolate neutrophils with classic polymorphonuclear phenotype from whole blood (14). A caveat is that some of these transcripts can also be expressed by monocyte-type cells, likely because they are involved in similar processes of innate immunity. A notable exception among the down-regulated transcripts is ZBTB16, which is considered a key marker of MAIT T cells but is decreased 1.57-fold in INOCA when most other MAIT-related transcripts were increased.

### Association of DEGs with prior GWAS or cardiovascular disease

An in-depth examination of the 199 INOCA DEGs via both manual and curated methods indicates that at least 18 of the transcripts have known associations with disease, especially cardiovascular and autoimmune disease (Table 4). While of unknown function, C1orf105 has been associated with heart failure, atrial fibrillation (Afib), and CAD by genome-wide association studies (GWAS) (15). Recently, complement factor H (CFH), which inhibits the consumption of complement component 3 (C3), was found to modulate inflammation and the atherosclerotic process in a mouse model (16).

**Table 4:** Transcripts associated by GWAS to cardiovascular or related diseases.

GWAS or disease related transcripts				Cell type	Pathway	Disease association	Annotation
Gene	Level	INOCA	CAD				
Symbol	(nRC)	Change	Change				
C1orf105	1.02	1.96	2.17	MAIT, neut, T	Unknown	Heart failure, Afib, CAD	Chromosome 1 open reading frame 105
CFH	38.53	1.54	1.23	MAIT T	Complement	Modulates atherosclerositic process in mouse	Complement factor H
CHIT1	14.30	−1.62	1.11	Macrophage, neut, MonoDC	TGF,	Athero!, biomarker heart failure, ALS, asthma	Chitinase 1
CRAT37	1.51	1.59	2.45	n/a		T2DM and HbA1C	Cervical cancer-associated transcript 37
DAAM2	100.53	−4.90	1.14	Neutrophil only	Wnt, regulated by hypoxia	Nephrotic syndome, eQTL, pulmonary ossification	Dishevelled associated activator of morphogenesis 2
GGT5	4.98	1.58	1.34	eos, baso, neut		aFib, hypertension in pilots	Gamma-glutamyltransferase 5
HTRA3	1.82	−1.99	−1.12	Neut and progen	Activation, proteolysis	GWAS LDL cholesterol	HtrA serine peptidase 3
IL18RAP	886.41	−1.51	−1.13	Neut, NK, MAIT, T cell	IL18 receptor	Atopic dermatitis, Crohn's, Lupus, MI Risk, Celiac	Interleukin 18 receptor accessory protein
LINC02009	6.32	−1.75	1.01	n/a	Links to CCR2, infected THP` macs	Osteoporosis	Long intergenic non-protein coding RNA 2009
LINC02506	3.66	8.30	8.69	WBC, heart	Expressed in heart	Obesity, Lipids, education level, smoking, height	Long intergenic non-protein coding RNA 2506
MPO	49.90	−1.73	1.09	Mono, neutrophil	Activation, free radical	Neutrophil granule, infection	Myeloperoxidase
PHF24	12.59	−2.00	−1.06	Neutrophil only	GABA signaling	GWAS to RA	PHD finger protein 24
PLIN4	175.57	−1.53	1.01	Neutrophil	Intracellular lipid droplets	Obesity, athero	Perilipin 4
SEMA6B	4.74	−2.19	−1.13	Neutrophil, mono	Cell recognition	GWAS mean corp vol	Semaphorin 6B
TMEM171	1.32	1.77	1.73	MAIT T, memT	TNF-a	Serum urate, FVIII	Transmembrane protein 171
TONSL-AS1	0.79	1.77	1.38	n/a		CAD	TONSL antisense RNA 1
TPST1	152.67	−1.55	1.14	Neutrophil, Baso, Eos	Wnt, CXCR4, FVIII hemostasis	CKD atherosclerosis, RA athero	Tyrosylprotein sulfotransferase 1
VNN1	346.12	−1.68	1.15	Mono, neutrophil	Hydrolyzes pantetheine to cysteamine	Sepsis, COVID progression, athero	Vanin 1
WSCD2	0.56	2.33	2.03	Treg, memB	Ferroptosis	Cardiometabolic Mexicans	WSC domain containing 2

Cell type abbreviations: MAIT T, mucosal-associated invariant T cell; Mono, monocyte; eos, eosinophil; baso, basophil; Treg, T regulatory cell; NK, natural killer cell; memB, memory B cell; pcDC, plasmacytoid dendritic cells; gdT, gamma-delta T cell; DC, dendritic cell; PMN, polymorphonuclear leucocyte/neutrophil.

Notably, the most down-regulated transcript, dishevelled-associated activator of morphogenesis 2 (DAAM2), has variants linked to the development of nephrotic syndrome, in which the renal microvasculature is disrupted (17). Both DAAM1 and DAAM2 are required for myocardial maturation (18), and the genetic variants are known to substantially modify RNA expression levels (19).

Also noteworthy is the transcript for myeloperoxidase (MPO), which is a well-characterized neutrophil granule protein involved in NETosis (20). MPO is considered a “high-risk” biomarker for patients with acute coronary syndrome (ACS), CAD, heart failure, hypertension, and stroke (21). MPO inhibitors are in clinical development for several cardiac indications (22), including heart failure with preserved ejection fraction (HFwPEF) (23). The most increased transcript LINC02506, has been associated by bivariate GWAS with the connection between obesity and serum lipid levels (24).

Several genes had prior associations to CAD or MI: C1orf105, CHIT1, IL18RAP, PLIN4, TONSL-AS1, TPST1, VNN1, and WSCD2. Others were associated with autoimmune disease, such as systemic lupus erythematosus (SLE), ALS, and RA: CHIT1, IL18RAP, PHF24, and TPST1. In several cases, there was noteworthy overlap between the CAD/MI and autoimmune associations: CHIT1, IL18RAP, and TPST1. Erythroferrone (ERFE, aka CTRP15) protein levels were elevated in 190 CAD patients vs. 70 controls (25). Interestingly, serum ERFE levels associate with mortality and cardiovascular event in hemodialysis and CKD patients in 2 cohorts (26).

### Self-organizing clusters of DEGs

**Figure 3:**
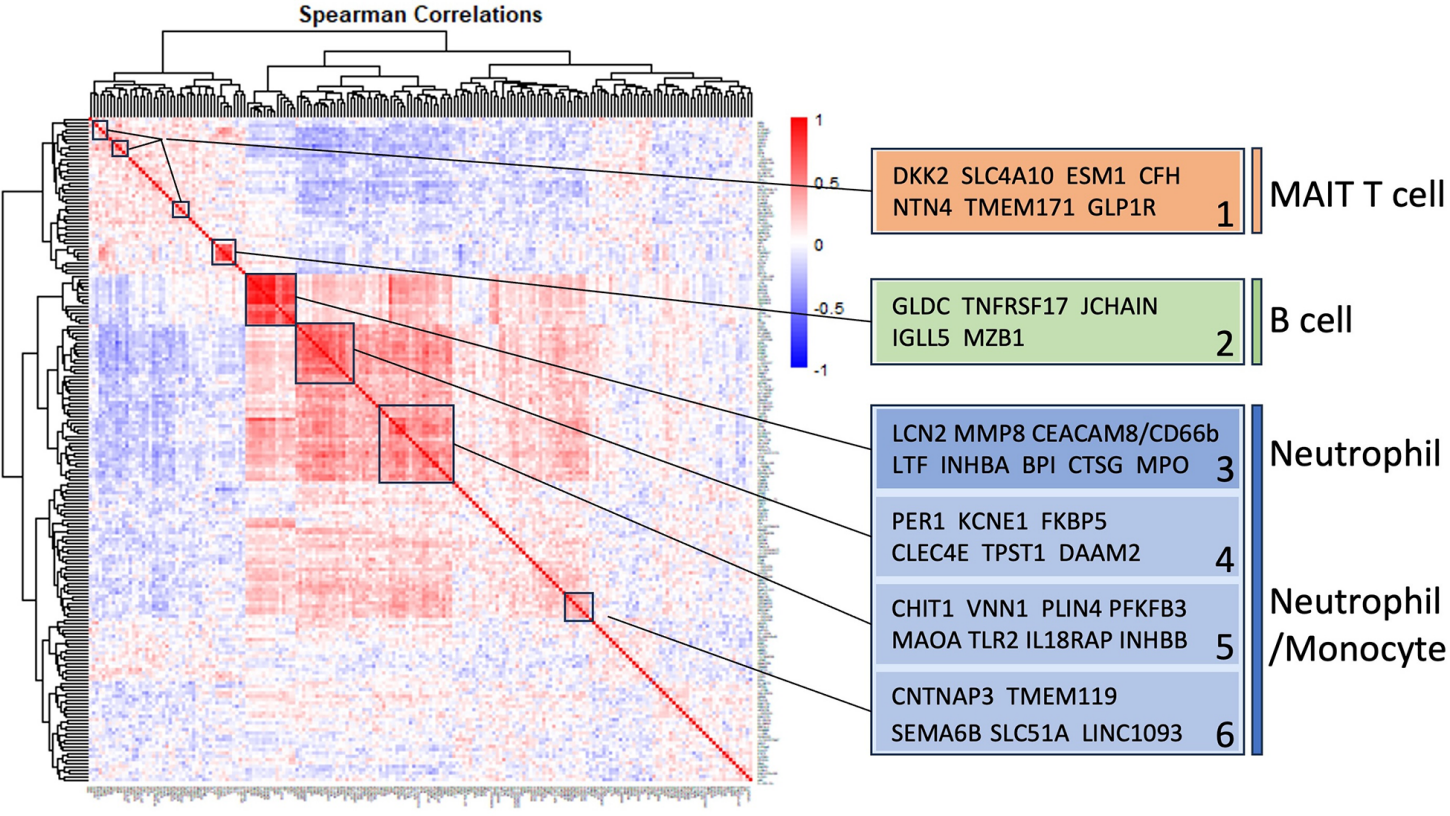
Self-organizing cluster analysis of DEGs in INOCA. The pattern of transcript changes in INOCA was analyzed by computing all possible Spearman correlations between the DEGs, and then conducting hierarchical clustering so that transcripts with similar patterns of expression across patients are shown in proximity (clusters) that are colored by a positive (red) or negative (blue) correlation. Certain clusters showed obvious associations with particular cell types, as shown on the right.

**Figure 4:**
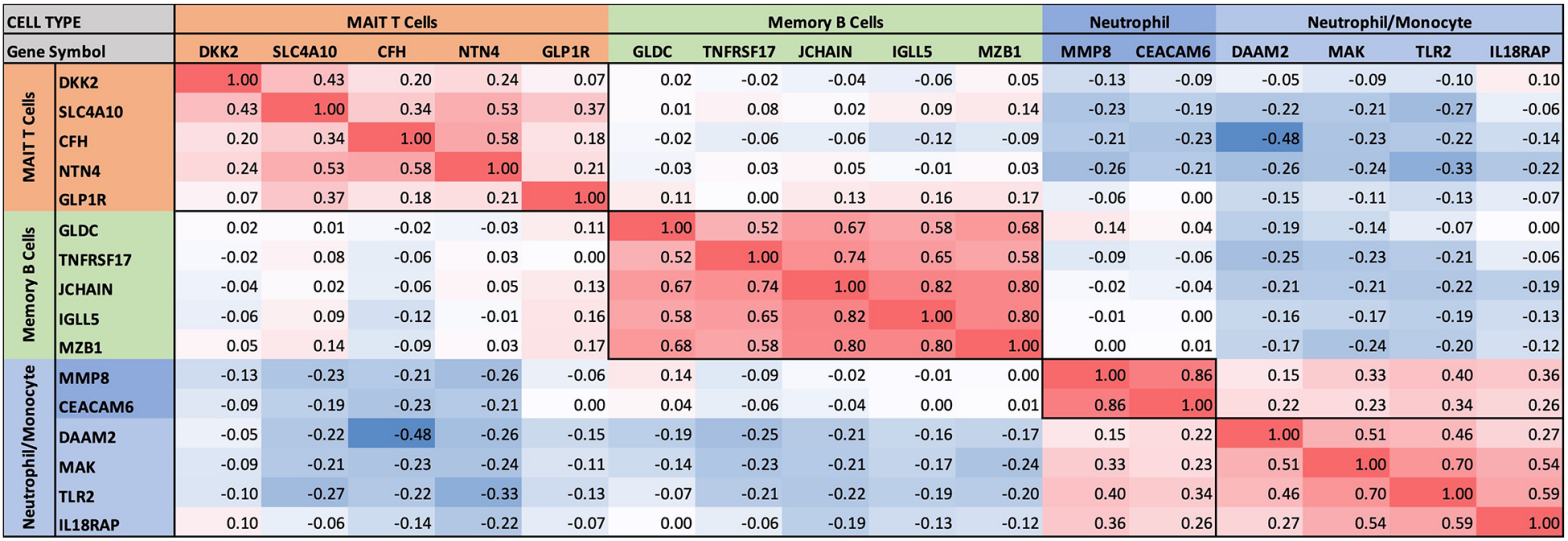
Intercorrelations of self-organizing clusters in INOCA patients. The hierarchical cluster from Figure 3 was constrained to show the major clusters and their interrelations at a resolution where the specific transcripts and numerical Spearman correlations are legible.

Pairwise correlations between all 199 INOCA transcripts, followed by hierarchical clustering, identified self-organizing clusters of transcripts that were interrelated (Figure 3). Initial analysis of these clusters identified patterns of their relationship to particular cell types, and key examples are highlighted for graphic clarity in Figure 4. A set of 3 clusters, including DKK2, SLC4A10, CFH, NTN4, and GLP1R, showed intercorrelations of up to 0.58, and were associated with MAIT cells.

A second tight cluster of transcripts, including GLDC, TNFRSF17, JCHAIN, IGLL5, and MZB1 showed intercorrelations of up to 0.82, and were strongly associated with memory B cells.

The largest cluster involved several well-known neutrophil-related transcripts, including a consensus marker for neutrophils, CEACAM8 (aka CD66b). Other well-known neutrophil transcripts, such as MMP8, TLR2, and CLEC4E were included in this cluster. Interestingly, the most strongly decreased transcript, DAAM2 was strongly intercorrelated in this cluster, with positive correlations to TLR2 (0.46), MAK (0.51), CLEC4E (0.66), and TPST1 (0.74). While leaning heavily toward neutrophils, many of these transcripts, such as TLR2, are also expressed, albeit at somewhat lower levels (∼50%) in monocytes, and thus clusters 4–6 are viewed as “neutrophil/monocyte” markers to indicate this uncertainty.

Thus, the transcripts organized themselves into groups that tracked the published associations of the transcripts or their proteins with particular cell types. While there were strong positive correlations within the clusters, there was a lesser, but consistently net negative correlation, up to −0.48 for CFH and DAAM2, between the MAIT cell cluster and the neutrophil cluster, as if there might be coordinated regulation (Figure 4). The cell types involved were pursued more directly with automated analysis using pre-curated data sets.

### Cell types involved in INOCA

The cell types associated with the 199 INOCA DEGs was assessed in an unbiased manner by 2 different approaches. There are extensive, pre-curated datasets of gene expression on flow cytometrically purified cell types (B cells, T cells, etc.), that is integrated with single-cell RNA sequencing (scRNAseq) data sets. Likely the largest and most extensively curated dataset is the Human Protein Atlas (HPA), which has an extensive subset of data directed to RNA transcript levels in purified and scRNAseq analyzed human immune cells (12). This dataset reports the level of any transcript across essentially all known cell types, and conversely, identifies the top transcripts that are unique to each of those cell types. This allows 2 complementary ways to analyze the cell types involved in INOCA.

#### DEGs associate with particular cell types

**Figure 5:**
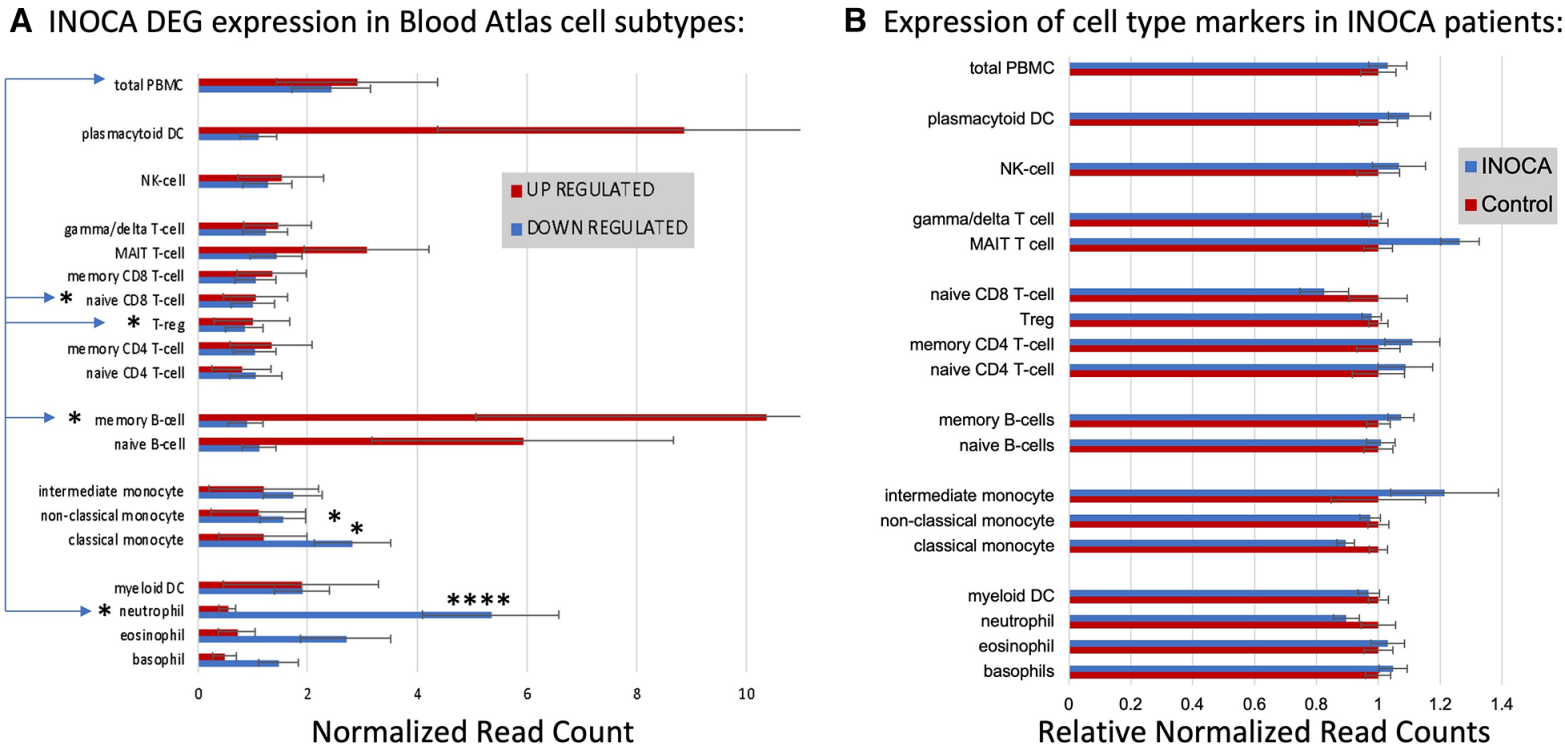
Cell type analysis of transcripts associated with INOCA. (**A**) INOCA DEGs were compared to a precurated expression databases (Human Protein Atlas) to find and graph their expression in the major blood cell types. The upregulated transcripts (red bars) were associated with plasmacytoid dendritic cells (DC), MAIT cells, and B cells, while the downregulated transcripts (blue bars) were mainly associated with granulocytes, especially neutrophils. (**B**) Regardless of the DEGs, the Blood Atlas was used to identify transcripts relatively unique to each cell type and then those transcripts were quantified and average between the groups to determine whether there was an overall shift in the abundance of those cell types, with generally no differences that could account for the changes in the DEGs.

First, the INOCA DEGs were cross-referenced to HPA to obtain the levels of each transcript in each of the circulating immune cell types curated in HPA: a total of 18 distinct cell types and the aggregated “total peripheral blood mononuclear cells (PMBC)” (Figure 5A, left panel, red bars). Because the upregulated and down-regulated transcripts seemed to associate with different cell types, they were considered separately. Of the 66 INOCA DEGs that were upregulated, 43 mRNAs could be mapped to the HPA dataset because ncRNAs, LINCs, and miRNAs are not well represented in HPA. The levels of upregulated transcripts were then averaged for each cell type to follow their pattern of expression across these known cell types. Comparing the average expression level (nRC) of upregulated transcripts between the aggregate “total PMBC” category (top), with each of the specific cell types mapped, it is evident that the upregulated transcripts were expressed most abundantly in plasmacytoid dendritic cells (pcDC), memory B cells, and naïve B cells, with the memory B cells reaching statistical significance vs. total PBMC.

Downregulated transcripts were similarly cross-referenced and averaged across cell types (Figure 5A, Left panel, blue bars). Downregulated transcripts (96 of 133 mappable in HPA) showed the highest expression in neutrophils, and somewhat lower expression in non-classical monocytes. Both the up- and downregulated transcripts showed significantly lower expression in naïve CD8T cells and T regulatory (Treg) cells. Thus, the DEGs are associated with particular cell types, but the question remains as to whether those cell types are somehow more or less abundant in INOCA, and therefore any transcript that they carry would be affected.

#### Cell-specific transcripts seem unaltered in INOCA

This question was addressed by a complementary analysis in which the DEGs are essentially ignored, and the HPA is used to identify transcripts that are highly enriched in a particular cell type. For example, the transcript for peptidase inhibitor 3 (PI3) is expressed 1,024-fold greater in neutrophils compared to the next highest cell type. A set of about 10 such enriched transcripts is reported by HPA for each cell type, and then the levels of those transcripts were computed in the INOCA data set. The particular RNA markers used to identify the blood cell types can be found in Supplementary Data S2. Thus, if a particular cell type is over- or under-represented in the patients with INOCA, then those enriched markers should reflect that abundance. As is evident in Figure 5B (Right), these cell-type enriched markers are not different between the INOCA and the Control patients, with no changes of greater than 20% up or down. By comparison, the DEGs are sometimes >10-fold (1,000%) different in a particular cell type. Other published markers for MAIT, mem B cells, and pcDC were also compared in the RNAseq results, but there was no systematic change in their RNA levels in the INOCA or CAD patients (Supplementary Data S3).

Thus, simply put, the DEGs are associated with particular cell types, but they are likely not altered because there are greater or fewer cells of that type in the blood. This differs from many prior observations about CAD-related DEGs, which is that they tend to associate with the Treg cell type (27, 28), and independently, flow cytometric analysis, and unique transcripts, indicate that there tend to be fewer Tregs in patients with CAD (29). Any analysis of cell types from RNA data, protein data, or flow cytometry is intrinsically limited by the fact that these markers can cross cell types. For instance, three markers JCHAIN/IgJ, IGLL5 and MZB1, which are strong markers for B-cells, are also markers of plasmacytoid cells in Crohn's Disease mesenteric adipose fat (30).

### Pathway analysis: the role of neutrophils

**Figure 6:**
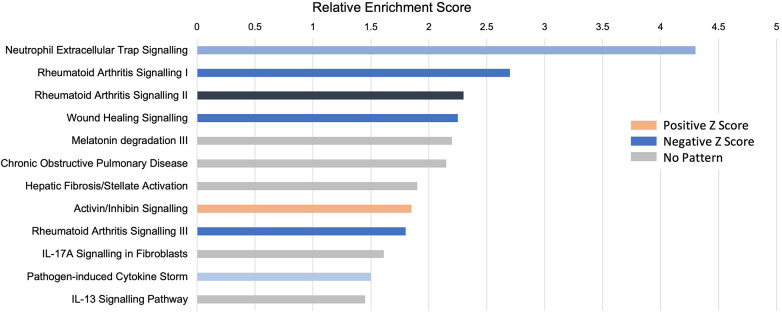
Pathway analysis of INOCA transcripts. The INOCA DEGs were submitted to automated, unbiased analysis of their association to specific biological pathways via Ingenuity Pathway Analysis (IPA). The results are shown as the relative overrepresentation (relative enrichment score, *X*-axis) in specific signaling pathways (*Y*-axis). The direction of the association is shown by the colored legend, with blue bars indicating that those transcripts were associated with the neutrophil extracellular trap (NET) pathway, which is downregulated in INOCA, thus generating a negative *Z* score.

An unbiased analysis of the biological pathways related to the DEGs was conducted in Ingenuity Pathway Analysis (IPA), confirming that the major pathways affected by INOCA were related to the “neutrophil extracellular trap signaling pathway” (-log_4.3_) and to a lesser, but still significant degree, 2 pathways related to rheumatoid arthritis signaling (Figure 6). The transcripts in this pathway are the family of transcripts reflected in Clusters 3–6 and having strong known relationships to neutrophil activation during innate immunity. The similarities with rheumatoid arthritis are intriguing, and will be discussed in depth later.

### Interferon pathway

IPA further identifies a disproportionate number of the DEGs as being related to the interferon signaling pathway (Table 5). By this automated analysis, 24 of the 199 INOCA transcripts are known to be modulated by or involved in the interferon (IFN) signaling pathway. In addition to the IFN-regulated transcripts shown in Table 5, the full 1,581 DEG list (not restricted by fold change) contain numerous transcripts indicative of IFN activation, especially FASLG, ICOS, IFIT1B, IFITM2, IFNGR2, IFNLR1, and IKZF2, and IKZF3 (which is increased in INOCA but decreased in CAD). IRF2 and IRF2BPL, IFNGR2, IFNE were all decreased. STAT4, a key IFN signaling mediator, was increased. Thus, there is a strong signal for activation of the interferon pathway in INOCA, a phenomenon that has been extensively studied in relation to CAD [reviewed in (31)].

**Table 5:** Interferon-related transcripts modulated in INOCA patients.

IFN response genes				Cell type	Pathway	Disease association	Annotation
Gene	Level	INOCA	CAD				
Symbol	(nRC)	Change	Change				
AGER	2.33	1.71	1.97	Low all	Inflammation, glucose	Diabetes, regulates T cell activation	Advanced glycosylation end-product receptor
CCL8	0.43	2.25	1.46	Monocyte, memB	Mono gran chemoattractant	IBS	C-C motif chemokine ligand 8
CFH	38.53	1.54	1.23	MAIT T	Complement	Modulates atherosclerositic process in mouse	Complement factor H
CLEC4E	760.50	−1.60	−1.03	Neutrophil, mono	Adhesion	AAA w KCNE1 MCEMP1!, STEMI biomarker	C-type lectin domain family 4 member E
ENTPD2	3.23	−1.60	1.12	Weak eos, neut			Ectonucleoside triphosphate diphosphohydrolase 2
ESM1	3.01	1.76	1.24	MAIT T, etc	Akt, VEGF	Immune signature in cancers	Endothelial cell specific molecule 1
FKBP5	1,263.81	−1.67	−1.05	Many positive	Immunosuppressor		FKBP prolyl isomerase 5
FN1	9.68	−2.18	3.39	Mono, DC			Fibronectin 1
GGT5	4.98	1.58	1.34	eos, baso, neut		aFib, hypertension in pilots	Gamma-glutamyltransferase 5
GPR83	1.44	−1.56	−1.10	Low all			G protein-coupled receptor 83
HRK	7.94	−1.84	−1.57	eos, memB			Harakiri, BCL2 interacting protein
IL17RB	1.70	−1.88	−1.17	Baso, Treg, Tcell	Interleukin 25 receptor	Inflammation	Interleukin 17 receptor B
IL18RAP	886.41	−1.51	−1.13	Neut, NK, MAIT, T cell	IL18 receptor	Atopic dermatitis, Crohn's, Lupus, MI Risk, Celiac	Interleukin 18 receptor accessory protein
INHBA	4.02	−1.99	−1.35	Neutrophil, mono	TGF-ß, differentiation	T cell suppressor	Inhibin subunit beta A, activin beta A-chain
LCN2	81.85	−1.53	1.05	Neutrophil	Transport, iron	Stroke, many others	Lipocalin 2
PFKFB3	732.93	−1.55	−1.01	Neutrophil mono	HIF, fructose, glycolytic	Hypoxia, sepsis,	6-phosphofructo-2-kinase
PIGR	0.85	−2.53	1.13	memB, B, neut	Exports IgA and IgM, mucosal		Polymeric immunoglobulin receptor
PSMB8	9.69	−1.55	−1.01	Modest all	Proteosome		Proteasome subunit beta 8 (from HGNC PSMB8)
RSPH14	5.52	−1.72	1.04	Neutrophil, mono	Unknown		Radial spoke head 14 homolog
SFRP1	1.11	−2.84	1.86	Low all	Wnt		Secreted frizzled related protein 1
TLR2	2,923.94	−1.51	−1.01	Neutrophil, mono	Toll like receptor	LPS receptor triggering activation	Toll like receptor 2
TMEM171	1.32	1.77	1.73	MAIT T, memT		Serum urate, FVIII	Transmembrane protein 171
TP73	1.52	1.77	1.60	T cell	Apoptosis		Tumor protein p73
ZBTB16	264.33	−1.57	−1.16	MAIT GDT	Antiinflamm, reg by lncANRIL	Con Ht Dis allele sp expr; PCOS; MS; AAA; met synd	Zinc finger and BTB domain containing 16

IPA analysis analyzed INOCA DEGs to identify enrichment for particular types of transcripts affected.

### Relationship of the INOCA signature to the CAD signature

In the full cohort of 177 patients, we had previously employed 2 different RNAseq platforms to identify a pattern of changes associated with CAD that is best characterized as decreased expression of T regulatory (Treg)-related transcripts (27, 28). To determine whether CAD and INOCA had similar transcripts involved, we compared the two lists. There is not strong overlap (7.8%) of the exact transcripts of the current INOCA DEGs with our prior analysis of CAD transcripts.

**Figure 7:**
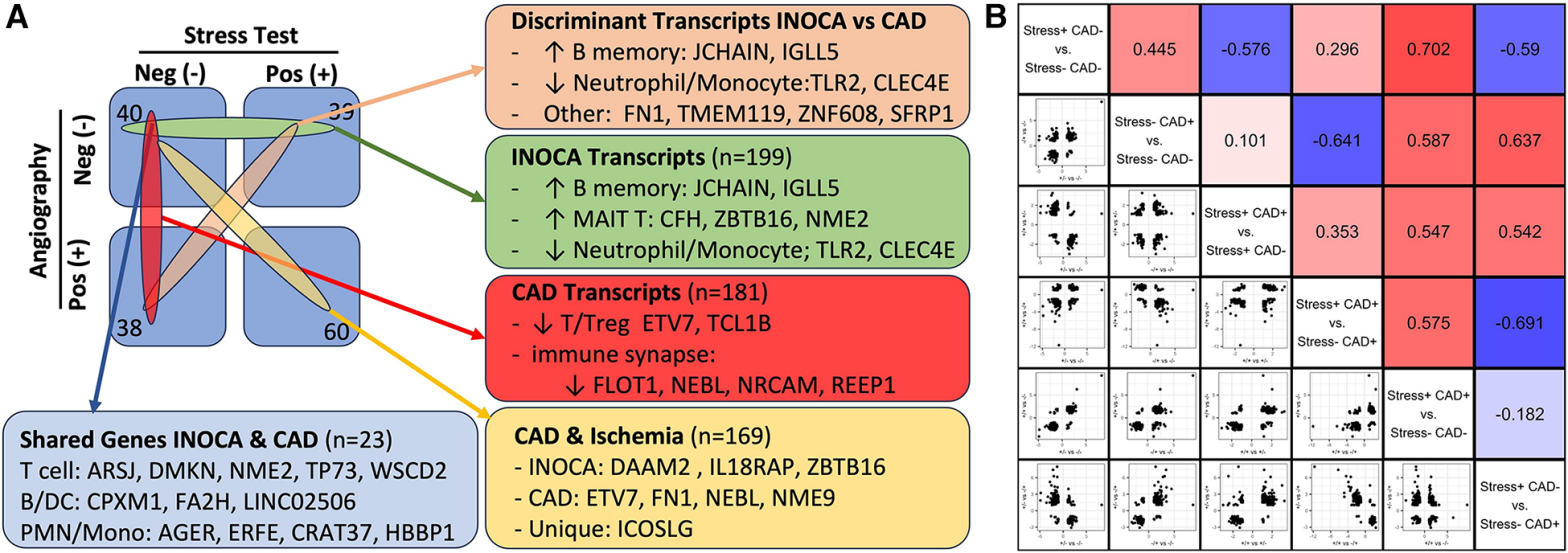
The relationship of INOCA DEGs to CAD DEGs. (**A**) The experimental design and *n* per group is shown in relation to the types of transcripts identified in INOCA vs. patients with angiographically detectable CAD, both compared to Stress(-)/CAD(-) group. (**B**) The matrix shows the correlation of DEG transcripts across the various groups in order to determine how INOCA transcripts, for instance, change relative to CAD and the other groups.

Within the current dataset, we conducted a similar analysis, but used the subgroups that are free of confounding ICA+ and Stress+ patients (*n* = 60), thus comparing Stress-/ICA- to Stress-/ICA+, to identify transcripts associated with obstructive coronaries in non-ischemic patients. The transcripts associated with CAD in Stress- patients yielded 181 transcripts. Both the transcript overlaps on DEGs (Figure 7A), and the correlation of the levels of the transcripts in the groups were compared (Figure 7B). This narrower definition of CAD identifies 23 transcripts that are also INOCA DEGs. While only ∼10% overlap, the odds of such an overlap occurring by chance are extremely small (*p* = 4.6 × 10^−22^). In all but 3 cases, the direction and magnitude of the change was similar in CAD and INOCA. The 3 exceptions, FN1, ZNF608, TMEM119, thus identify discriminant gene expression between CAD and INOCA.

Because transcripts could change in a similar manner in CAD and INOCA, but miss the DEG thresholds, the pooled list of CAD and INOCA DEGs was correlated between their expression when grouped by CAD vs. grouped by INOCA (Figure 7B). In this analysis, there is a net positive correlation of 0.44, which likewise indicates some shared properties of CAD and INOCA. It is striking that the LINC02506 transcript, which is the most elevated transcript in INOCA (up 8.3-fold), is also the most elevated transcript in this sub-analysis of CAD (up 8.7-fold). All possible correlations between the transcripts in the subgroups are shown in Figure 7B.

### Infection-related transcripts

The recurring appearance of neutrophil-related genes suggested that INOCA could be related to some type of active infection. In prior publications, we identified neutrophil-related transcripts that respond to (1) free floating “planktonic” infections, such as pseudomonas in the lungs (32), (2) biofilm infections, such as those occurring in the appendix or on prosthetic joints (32), and (3) viral infections, such as SARS-CoV2 (33). Using these biomarkers, and other known transcripts such MPO and CTSG, we computed their expression levels (nRC) in the 4 major groups, and see no evidence for any type of acute infection in these patients. Although some patients had elevated values, there was no pattern by group (Table 6). The INOCA DEGs, CEACAM8, CTSG, and MPO are visibly lower in the INOCA group, but do not pass the non-parametric test used here, which differs from the DeSeq2 analysis that identifies DEGs. Thus, we can exclude an active infection as the cause of INOCA, but cannot exclude a prior infection that might have triggered a more stable immune defect.

**Table 6:** Pre-defined transcripts associated with infections and their association with INOCA or CAD.

Gene symbol	Description	Biomarker type	CAD negative		CAD positive	
			Stress negative (*N* = 40)	Stress positive (*N* = 39)	Stress negative (*N* = 38)	Stress positive (*N* = 60)
			Control	INOCA	CAD + Str-	CAD + Str+
ACTB	Actin-ß	Control	26,300 (7,520)	24,500 (3,940)	27,000 (4,910)*	25,400 (6,030)
DEFA1 *	Defensin-a1	Bacterial	1.96 (3.41)	1.55 (2.00)	3.92 (8.13)	2.00 (2.41)
MPO	Myeloperoxidase	Bacterial	60.5 (108)	35.1 (31.6)	66.0 (85.9)*	52.2 (52.9)
CTSG	Cathepsin G	Bacterial	10.6 (18.5)	6.30 (8.71)	12.9 (20.3)	9.67 (11.1)
BPI	Bacteriacidal Permeability Increasing	Bacterial	63.4 (83.4)	38.1 (26.2)	73.0 (111)	55.4 (56.9)
ALPL	Alkaline Phosphatase	Biofilm	1,200 (1,510)	952 (756)	1,210 (1,080)	951 (953)
CXCR2	Interleukin 8 Receptor ß	Biofilm	5,650 (4,430)	4,480 (2,260)	5,510 (2,570)	4,430 (1,650)
IFI27	Interferon Inducible Protein 27	Viral	0.0239 (0.151)	0 (0)	0.0495 (0.305)	0 (0)
RSAD2	Radical S-Adenosyl Methionyl Domain 2	Viral	316 (354)	322 (348)	680 (1,650)	464 (698)
CEACAM8	CD66b/CEA Cell Adhesion 8	Neutrophil	71.2 (111)	43.9 (45.4)	78.4 (124)	62.6 (80.6)

Selected transcripts with prior evidence for biomarker activity for infections are reported in normalized read counts (nRC) from DeSeq2 analysis. DEFA1 values are derived from a different alignment, due to anomalies in the STAR aligner settings that exclude genes with high copy number variants. Values are mean (s.d.) expression level.

* Indicates *p* < 0.05 uncorrected vs. INOCA group.

### Sex and age effects on INOCA-related transcripts

**Figure 8:**
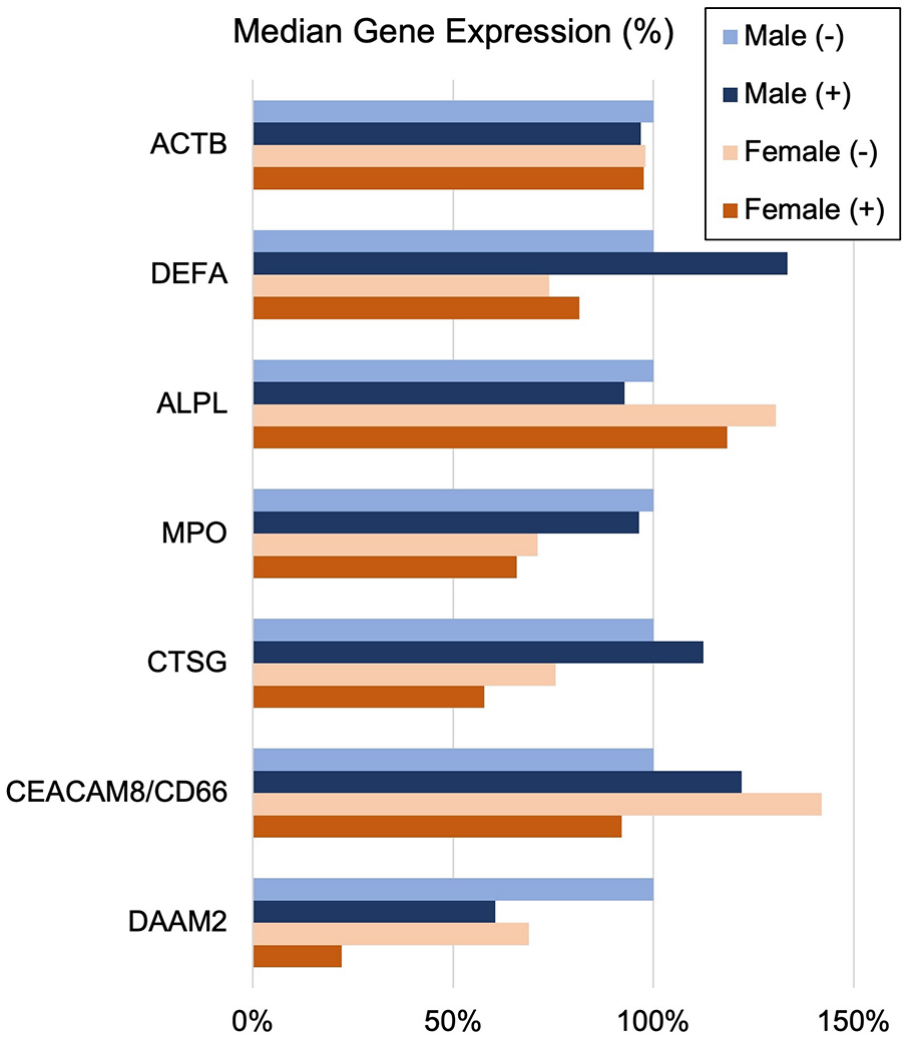
Sex-related changes in INOCA and unrelated neutrophil transcripts. The sex-related expression of the INOCA-related transcripts CEACAM8, CTSG, DAAM2, and MPO were calculated as a function of sex. For comparison, other unrelated transcripts expressed across all cell types (ACTB), or relatively specific for neutrophils, but not related to INOCA (DEFA1, ALPL) are also calculated as a function of sex (Male vs. Female) and INOCA status (no INOCA = (-), INOCA = (+)). Expression levels are median nRC relative to the percent of the male no INOCA group.

The often-described increased prevalence of INOCA in females warranted analysis of INOCA transcripts as a function of sex. As shown in Figure 8, most transcripts do not differ as a function of sex, as exemplified by the reference example of actin B (ACTB). However, by contrast, the INOCA-related transcripts, MPO, CTSG, CEACAM8 and especially DAAM2, tended to be lower in females than males, and showed a greater change in the INOCA patients. Likewise, DAAM2 and CEACAM8 tended to be more elevated in patients over the age of 65 vs. under, but the difference was not statistically significant (not shown).

## Discussion

**Figure 9:**
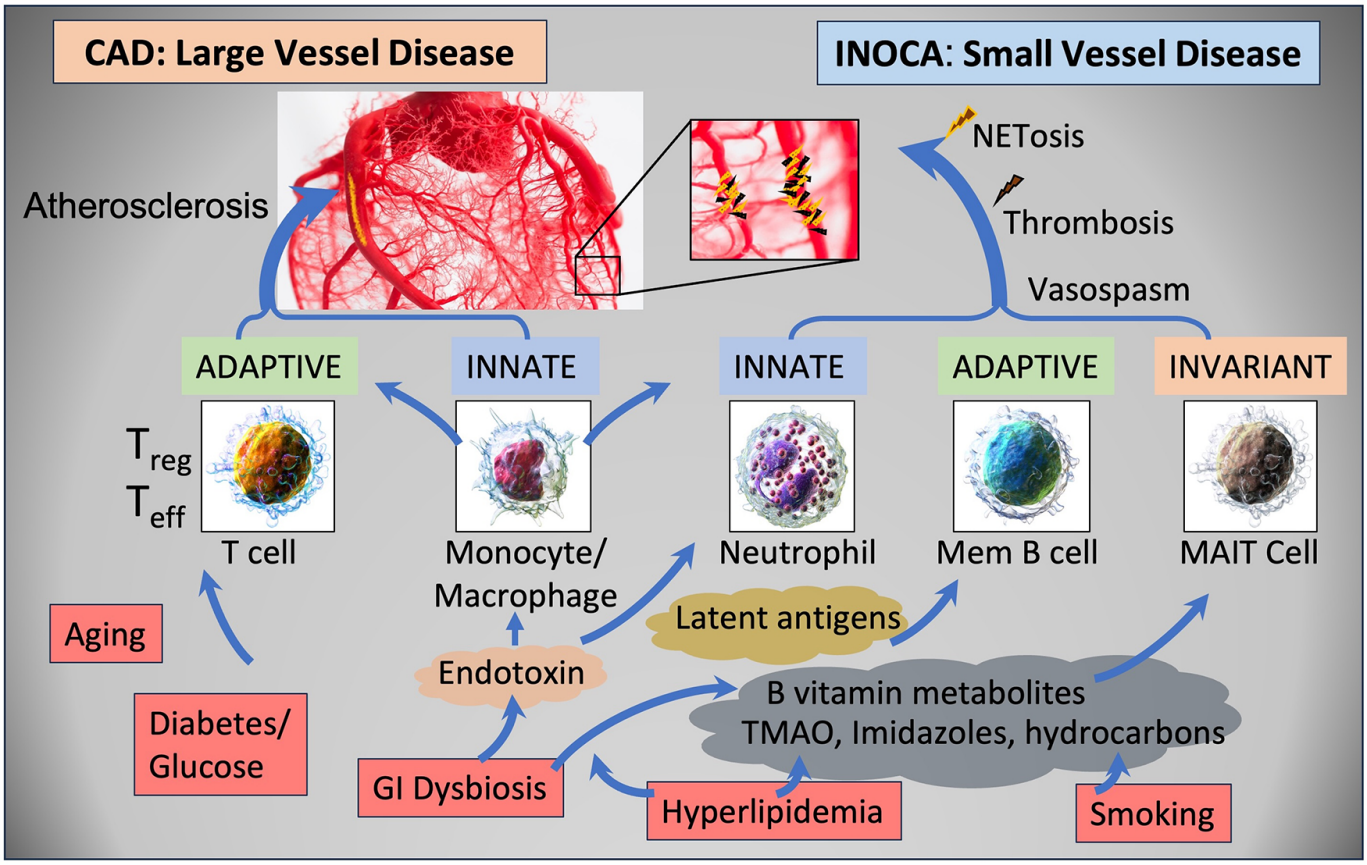
Schematic summary of the transcripts, cell types, and pathways involved in CAD and INOCA. CAD and INOCA principally differ in which vessels, large or small, are affected. At the level of the transcriptome, CAD is associated with changes in RNAs associated with T cells, especially Tregs, and this difference is extensively confirmed by overt changes in Treg abundance in CAD. INOCA is associated with a transcriptome pattern reflecting changes in transcripts related to mucosal-associated invariant T (MAIT) cells, memory B cells, and neutrophils. An interesting hypothesis is that GI dysbiosis, hyperlipidemia, and hydrocarbons could lead to activation of this “invariant” arm of the adaptive immune system, thereby causing microvascular disease.

The current results raise a number of interesting possibilities about the etiology of INOCA, with the most striking relationship being to a possible autoimmune microvascular dysfunction. A high-level graphical summary of the results is shown in Figure 9, to help visualize the potential relationship between CAD, INOCA and the particular RNA expression patterns related to known cell types.

### The RNA signature and cellular components of INOCA

The increased expression of select genes related to B cell, pcDC, and MAIT cell activation, combined with decreased levels of neutrophil transcripts is an interesting and unique signature in blood RNA. The involvement of MAIT cells is highlighted by changes in several transcripts, including ZBTB16, aka PLZF, considered the master regulator of MAIT cells (34). It may be important that ZBTB16 transcript is decreased while almost all other MAIT associated transcripts are increased. MAIT cells have an intimate relationship to the gut and other microbiomes because they possess an invariant T Cell Receptor (TCR) that is tuned to bacterial metabolites, especially of B vitamins, that are presented on the MHC-related MR1 (35). MAIT cells would normally perform innate-like control of bacteria, but they are known to cross-stimulate other cell types such as B cells and conventional T cells in some autoimmune conditions (36). MAIT cells comprise about 5% of circulating CD3+ cells and have multiple influences on autoimmune diseases (34). For example, MAIT cells are chronically activated in patients with autoimmune liver disease (37). Interestingly, MAIT cells are known to be reactive against polyfluoroalkyl substances (PFAS), so called “forever chemicals” (38), and are functionally affected by smoking and COPD (39). Neutrophils can suppress MAIT cells, but conversely, activated MAIT cells can produce high levels of TNF-α that can induce neutrophil apoptosis (40), suggesting a generally antagonistic relationship between these cell types. This would be consistent with the net negative correlations in gene expression between the MAIT transcripts and neutrophil transcripts in Figure 4. The reduced neutrophil-associated transcripts that are seen in INOCA are not observed in the CAD patients (Table 3), and differs from published associations of NET-related activity with CAD (41). However, the relationship between NET activity and neutrophil RNA levels is quite complicated, but suggestive of the potential involvement of NETosis in INOCA, as discussed below.

### Potential immune triggers for an INOCA RNA profile

The history of atherosclerosis research is punctuated with a variety of viral, bacterial, and autoimmune initiators for adverse vascular remodeling. While almost every virus and bacteria has been associated with atherosclerosis, recent studies on influenza and SARS-CoV2 (SCV2) lend very credible support to the likelihood of viral initiation of immune changes that lead to myocarditis and other cardiac diseases. Large studies have demonstrated a temporal association between influenza infection and myocardial infarction (42). Likewise, flu vaccines have a provable benefit in randomized control trials at preventing adverse events in high-risk CV patients (43).

The COVID-19 pandemic highlighted the connection between viral infections and adverse CV events. A signature event in SCV2 infection was an increased neutrophil to lymphocyte ratio (NLR) that led to striking NETosis and end organ damage in ICU patients (44). This was detectable in blood RNA as marked elevation of neutrophil transcripts, such as MPO, DEFA1, and neutrophil elastase (ELANE), but decreased levels of T cell transcripts (33). The data on SARS-CoV2 vaccines is more complex, with some evidence that RNA-based spike protein vaccines triggering myocarditis in some patients, but showing an overall protective effect on cardiovascular health in elderly “at risk” patients (45).

Recent sequencing of bacterial 16S rRNA in whole blood suggest that there are detectable changes in patients with acute coronary syndrome (ACS) (46). Changes in the gut microbiome have been associated with autoimmune inflammatory arthritis, and coincidentally affect B vitamin metabolism (47). Likewise, an increasing literature suggests that microbiome changes affect CAD progression via other metabolites such as imidazole propionate (48), TMAO, and short chain fatty acids such as propionate or butyrate [reviewed in (49)].

Regardless of the initiating event, the INOCA RNA profile includes many transcripts that would be potent immune modifiers. In a very striking coincidence, complement factor H (CFH, increased 1.5-fold in INOCA and 1.2–1.46 in CAD groups) was recently described as important to the atherosclerotic process in mouse models, and present in human atherosclerotic lesions (16).

### Potential autoimmune component to INOCA

Changes in the acquired (B cell and pcDC), invariant (MAIT T), and innate (neutrophil) immune system describe an immune dysregulation that parallels other known autoimmune syndromes such as lupus/SLE, systemic sclerosis, rheumatoid arthritis (RA), and others. Patients with SLE have more severe coronary microvascular disease (CMD) than symptomatic matched controls (50). Patients with RA have ∼1.5-fold increased risk of cardiovascular disease attributed to chronic inflammation (51). In women with CMD, about 10% have autoimmune rheumatic diseases (52). Further, disease-modifying anti-rheumatic drugs are showing beneficial effects on cardiovascular risk (53).

The pathway analysis shown in Figure 6 identified 3 pathways related to RA signaling that are also affected in INOCA. Those pathways involve 3 collagen genes (COL13A1, COL17A1, COL9A2), DKK2, FN1, interleukin receptors (IL17RB, IL18RAP), J chain, MMP8, SFRP1, and TLR2. The IL17 pathway is especially interesting therapeutically because it is a target of approved immunotherapies for psoriasis, which is an autoimmune disease with strong CV comorbidity (54). Two transcripts, JCHAIN (IgJ) and TNFRS17, both increased 1.7-fold and with high absolute expression level, have previously been identified as markers of plasmacytoid cells in autoimmune disease (55). Other data indicates that JCHAIN/IgJ is a marker of “plasmablasts” cells that are IgM and IgA secretors (56). A third gene, IGLL5 lambda chain, is a known marker of RA (57). IL18RAP is associated with a variety of autoimmune diseases including lupus, celiac, but especially with CAD via whole blood expression levels (58), and associated with MI via IL18 SNPs (59).

While similar to SLE, certain aspects of the INOCA pattern, however, are different. For instance, SLE has typically been thought to involve low density granulocytes (LDG) (60). However, RNA markers of LDGs, such as RSAD2, MX1, and IFI44 (60), are unchanged in INOCA. Rather, in INOCA the changes appear to be limited to more conventional granulocytes, especially the neutrophils. Antiphospholipid syndrome (APS) also exhibits cardiac manifestations consistent with microvascular disease and INOCA (61). However, the present data tends to discount APS because we previously saw strong HLA association in aspirin resistance (62), which is similar to APS, and yet we do not see HLA changes in the present studies.

### From autoimmunity to microvascular disease

It is accepted that innate immune cells, especially neutrophils, and unconventional T cells such as γδ T cells and MAIT cells are involved in microvascular remodeling, most notably in hypertension (63). Systemic sclerosis, which is caused by autoantibodies to anti-centromere proteins or anti-topoisomerase antibodies, among others, has well documented effects on the microvasculature (64). A systemic inflammation index, computed as the platelet count × neutrophil/lymphocyte ratio (NLR), is elevated in patients with microvascular dysfunction associated with cardiac syndrome X (CSX), which is ANOCA/INOCA-like (65). Recent pilot data suggests that NET markers in blood increase the predictive ability for all-cause mortality in patients with cardiac arrest (66).

Colchicine, a microtubule assembly inhibitor, in low doses has recently been approved to treat CAD, and its proposed mode of action is anti-inflammatory. Colchicine has known inhibitory effects on NET formation in the heart post-MI and in patients with ACS (67). Colchicine has a preferential effect on neutrophils because they generally lack the P-glycoprotein efflux pump, and thus inhibits intracellular mobilization and release of granule contents, especially elastases and defensins [reviewed in (68)].

### The relationship between INOCA and CAD

A different perspective on the current data demonstrates that INOCA and CAD have both shared and unique RNA profiles. While only ∼10% of the transcripts are identical between the 2 disorders, the overall correlation of the transcripts between the two groupings of the dataset shows a net positive correlation of 0.45. An important consequence of this correlation is that prior analyses might have missed important changes in CAD. The presence of the INOCA patients in what was previously considered a “control” group for CAD, had the effect of masking some transcripts from being detected. For instance, LINC02506 is up 8.3-fold in INOCA, and up 8.7-fold in CAD, when the stress+ patients are omitted. However, in our prior combined analysis of the same patients that included stress+ patients and were grouped only by CAD, at the same 20% threshold, there is a much more modest 2-fold increase in LINC02506 levels. Thus, the presence of the INOCA patients in the “control” group of patients with stenosis <20% is a major confounding factor in identifying CAD-related gene expression. This is further complicated by the known potential for significant CAD of larger arteries but that is extra-luminal and thus non-obstructive. This will require a much more stringent definition of “controls” for future biomarker analysis of CAD.

### Study limitations

The size of the study is quite powerful for a discovery-type study to generate hypotheses about the underlying etiology of INOCA. The analytical methods are diverse and provide a relatively consistent picture of activation of mem B, pcDC, and MAIT T cell transcripts, with decreased levels of neutrophil transcripts.

A significant limitation is that the clinical syndrome of INOCA is imperfectly measured by a stress test. In general, stress tests are prone to “false positive” results that can lead to unnecessary angiography, and thus, it is likely that some patients classified as INOCA in the present analysis are, in fact, not ischemic. There are a variety of more sophisticated tests, such as coronary flow reserve and acetylcholine provocation test during angiography, that could be used to measure microvascular function, but because they are not in routine use, this would require more costly invasive testing.

While the cohort is by far the largest RNAseq analysis of CAD and INOCA to date, there are intrinsic limitations to measuring 28 K transcripts in 177 subjects. Even a low noise level creates false positives, and negatives, that cannot be statistically corrected.

Another limitation is that while RNA can be quite accurately quantified by RNAseq, the RNA level has an unpredictable relationship to the encoded protein levels. In some cases, RNAs can be elevated because they are not being translated, and thus protein expression can be low. Conversely, it is known that many RNAs are degraded in the process of translation, and thus a reduced RNA level can be associated with elevated protein levels. Ideally, isolated neutrophils would be used for semi-quantitation of select protein targets to determine how the RNA levels related to protein levels. Further, functional analysis of NETosis and interferon pathways would be used to validate the RNA findings.

In retrospect, an in-depth cell type analysis by flow cytometry would have been helpful to evaluate the potential role of changes in the abundance of mem B cells, MAIT cells, and neutrophils, with only the latter being evident from a conventional laboratory blood count. However, this is quite difficult to accomplish in a busy cath lab setting. Furthermore, many prior studies have only documented changes in Treg cell abundance in CAD, though we are not aware of a similar analysis in INOCA.

## Conclusions

INOCA is associated with elevated levels of transcripts relevant to the acquired immune system, especially memory B, pcDC, and MAIT cell lineages, and a reciprocal decrease in transcripts associated with the innate immune system, particularly neutrophils.

The INOCA profile has significant differences with the CAD profile. The INOCA transcripts affected are of relatively high absolute abundance and some have high fold-changes compared to the relatively low level and low fold changes observed for CAD.

The inclusion of INOCA patients in prior “control” groups for CAD likely masked some significant transcripts that are shared by both INOCA and CAD.

Based on the expression profile, despite the innate immune system involvement, INOCA likely does not involve an active bacterial, biofilm, or viral infection.

The RNA profile is consistent with an indolent autoimmune syndrome, leading to chronic, low-grade activation of NETosis, with one of the targets being the coronary microvasculature.

### Future directions

The present results strongly indicate that future studies of CAD biomarkers in blood should carefully account for INOCA patients. The presence of small vessel disease will likely complicate any biomarker analysis.

An interesting question is whether the inflammatory transcript profile described herein would correct itself during treatment with colchicine, RA treatment or psoriasis immunotherapies. If so, the RNA profile could be used to track the efficacy and possibly dosing of these therapeutic modalities.

## Data Availability

The datasets presented in this study can be found in online repositories. The names of the repository/repositories and accession number(s) can be found below: https://www.ncbi.nlm.nih.gov/geo/, GSE221911.

## Supplementary material

Supplementary Data S1 Complete list of 199 DEGs related to INOCA with annotation.

Supplementary Data S2 Cell-specific markers used, and their individual expression on control vs. INOCA.

Supplementary Data S3 Additional published markers of MAIT, pcDC, and mem B cells.
